# The Relations of Dental Diseases to General Diseases

**Published:** 1899-10

**Authors:** William Hunter


### Abstracts and Translations.

### THE RELATIONS OF DENTAL DISEASES TO GENERAL DISEASES.

BY WILLIAM HUNTER, M.D., F.R.C.P.

(Continued from page 522.)

### RELATION TO GENERAL INFECTIONS AND INFECTIVE GASTRITIS.

THE class of diseases whose relation to dental diseases is not so clear and possibly hardly less important are (1) those of obviously infective nature, the only question being the channel by which the infection has entered the body,—such affections as acute osteomyelitis and necrosis occurring apart from injury; empyema, meningitis, ulcerative endocarditis, some forms of acute nephritis; and (2) the whole series of cases of obscure nature probably infective, characterized generally by features of blood-poisoning, and probably determined by some general infection, the origin of which cannot be made out.

When, as sometimes happens, such cases can be traced to infection from diseased teeth, light is cast upon the whole subject of the *rôle* possibly played by dental diseases in many general conditions. One of these conditions I consider to be *subacute and chronic infective gastritis.* I cannot better illustrate the class of case I refer to than by describing to you a case recently under my observation.

The case was that of a lady, aged sixty-two, sent to me by Dr. Ferris, of Uxbridge, where she had been on a visit, with a history of having been ill for nearly a year; symptoms all referring to stomach, pain, sickness, and obscure abdominal distress, necessitating use of opium; loss of flesh; symptoms suggesting cancer of stomach, although no signs of growth could be detected.

She presented a wasted, somewhat cachectic appearance, with pale, sallow complexion.

Next to the gastric pain, intermittent in character, she complained mostly of a constant bitter taste in mouth, a great loathing and distaste for food, an inability to taste her food, and a sense of nausea with occasional sickness, the sickness having no relation to food, coming on usually in the morning.

On examination I could not find any sign of malignant disease in abdominal or pelvic organs. The only physical signs of disease were to be found in the mouth. Her teeth had all been removed with the exception of four stumps. She wore two plates of false teeth, which were scrupulously clean. The gums, except around the four stumps, were quite healthy. These stumps presented a dark, rotten, dead appearance, and, in the case of three of them, on pressure pus welled out freely from their sockets through small pouting sinuses.

This condition of teeth she had had, she said, for a year or more.

The tongue presented a moist, soft, flabby appearance.

The provisional diagnosis I made, after full consideration, was against it being cancer, and in favor of the gastric condition being due to continual swallowing of pus. The sickness she complained of appeared to be an utter loathing of food, with nausea, rather than actual sickness, and these might be well accounted for by the quantity of pus she was constantly sucking into her mouth from the decayed stumps.

I directed her to have the stumps removed at once, preparatory to any further treatment.

She consented to this after some demur (the stumps, she said, could not possibly be at fault, as they had been in that condition for a year or more,—a period closely corresponding, it will be noted, to the duration of her illness), and had them removed the following day, apparently with the most immediate and satisfactory result, for when she reported herself a week later, she had had only one return of sickness and gastric pain,—viz., the day after the extraction; had lost all bad taste in mouth; was able to taste her food for the first time for months; the tongue now quite clean.

This striking improvement, however, did not last. Three days later the gastric pain, with sickness and vomiting, recurred, and continued on and off for the next two weeks. A specimen of the vomit was obtained at this time,—*i.e.,* three weeks after the extraction of the teeth; it was of a somewhat brown color, with rusty, and here and there redder, streaks, and small flakes resembling bits of grape-skin.

On examination I found these flakes to consist of fibrinous exudation, with numerous leucocytes and crowds of streptococcus organisms, along with bacilli and cocci in much smaller number.

As it was now three weeks since the suppuration in the gums had been stopped, I regarded the presence of these organisms as confirming my original suspicion,—viz., that the case was one of subacute infective gastritis or subacute infective catarrh.

Up to this time the patient had been struggling to get about, but she was now too ill to do so.

The subsequent course of the case was as follows: I confined her to her bed for eleven days, fed her entirely on peptonized milk and gruel, beginning with one and a half pints daily, applied counter-irritation to stomach with sedatives internally; and, specially to combat the streptococcal infection, gave salicylic acid in three-grain doses, thrice daily, after food.

The improvement was immediate and continuous. The sickness and pain were entirely checked from the first day, the tongue lost its raw, angry look, and became normal. The pulse fell from 102 to 80 and 70. Temperature normal (which had been 99° F.). In ten days' time she was able to go out driving and to return home to the West of England. I kept her on peptonized milk and gruel for another month, at the end of which time she reported herself as still without return of pain. A month later she reported herself still free from any pain, and gaining in weight, although still on milk diet. It is now five months since her illness, and she has no recurrence of any stomach trouble.

Cases of this kind could, I have no doubt, be paralleled by many others in the experience of members of the society. One almost identical is recorded by Miller (p. 298). A lady, aged forty-five, complained for months of severe pains caused by eating, loss of appetite, indigestion, etc.,—her troubles so great that she declared life had become insupportable. She showed two envelopes filled with prescriptions both for internal and external use. A glance into her mouth, its fetid odor, and the inflammation and suppuration of gums suggested at once the cause. The cleansing of the mouth and the use of antiseptic and astringent mouth-washes caused such a pronounced improvement in a fortnight that the patient could not often enough express her thanks.

In this case, he adds, the source of the trouble was so apparent he could not understand why it had not been discovered before. And it is with reference to the constant overlooking of such class of cases that he elsewhere brings the charge against many physicians, that "the custom to disregard dental diseases altogether as a factor in pathology is as unjust to their patients as it is discreditable to their profession. No physician can afford to be without a thorough knowledge of the pathological processes occurring in the human mouth, and their relation to general diseases."

INFECTION FROM THE MOUTH AS A CAUSE OF INFECTIVE GASTRITIS.

While resembling, however, many others of a like kind, the case I have recorded presents some special points of interest: in respect, namely, of the actual proof of infection of the stomach by streptococcal organisms,—viz., the presence of pus, fibrin, and leucocytes, and numerous pus organisms three weeks after removal of source of the infection.

That organisms play a pathogenic *rôle* in stomach troubles by setting up fermentative troubles is fully recognized.

Most writers on the subject would appear to regard this as the only way in which gastric disorders may arise from organismal infection. The case I have recorded would appear to suggest in certain cases another and even more intimate connection between gastric disorder and organismal invasion,—viz., by an actual infection of the mucosa, and the setting up of, at first catarrh, and subsequently of more permanent inflammatory changes in the mucosa and submucosa.

The liability to such infection will be the greater if the organisms introduced be the recognized pyogenic organisms setting up inflammation and suppuration elsewhere.

Now the number of organisms that enter the stomach with the food and from the mouth is very large, and a very considerable proportion of these are permanently to be found in the stomach contents. Out of twenty-five kinds of bacteria found by Miller in the human mouth, eight were found in the stomach contents.

Moreover, the old view that organisms entering by the stomach were destroyed by the gastric juice has had to be abandoned in the light of the observations of Macfadyen and others.

Only a certain proportion are destroyed. From Miller's experiments the conclusion is warranted that all bacteria swallowed at the beginning of a meal may pass alive into the intestine (in the fæces he found twelve of the twenty-five mouth organisms). It is only when the acidity of the gastric juice is considerable—say an hour or two after meals—that it exercises any direct bactericidal action.

"If we, furthermore, take into consideration the various and numerous affections in which the quantity of gastric juice, its percentage of hydrochloric acid, is abnormally small, it will appear as though the stomach can afford almost no protection whatever against the passage of pathogenic micro-organisms through into the intestinal canal," or, indeed, under favorable conditions, to their sojourn in the stomach itself.

If, in addition to diminished acidity, we have also increased supply of pathogenic organisms, and, moreover, the conditions be such that these organisms reach the stomach not only with food, but at all times, in the intervals between digestion as well as when food is taken, we have, I consider, pre-eminently favorable conditions for not only a temporary sojourn, but for possibly a permanent infection.

Such are the conditions typically presented in many cases of dental disease,—viz., long-standing suppurating conditions around teeth and gums, constant swallowing of pyogenic organisms, impaired digestion with diminished acidity.

That under such circumstances increased fermentations go on we know.

What, however, is not recognized, what certainly I never realized till I made the observations just described, is that there may be not only increase of the fermentative processes, but also, what is more dangerous, that organisms with well-defined pathogenic properties may become, so to speak, permanently established in the stomach.

The mucosa of the stomach remains permanently exposed to infection from pyogenic organisms, and may in time become actually infected with them.

The subacute and chronic catarrh so often met with in association with suppurative dental disease may thus be, as in the case I have recorded, of infective origin, not merely the result of irritation set up by fermentation of food products, but the result of definite invasion of the mucosa and submucosa.

Under certain circumstances it is conceivable that the effect might not stop short at catarrh, but, on the contrary, lead to subacute inflammatory changes, resulting, as all such changes in glandular organs do,—viz., in atrophy of glandular cells and increase of fibrous tissue.

The condition termed *atrophy of the mucous membrane of the stomach,* so well studied by Dr. Samuel Fenwick, and the more acute inflammatory change occasionally met with and likened by him to *eczema of the stomach,* may thus be, and in my opinion probably are, the result of old-standing infections.

### PHLEGMONOUS GASTRITIS.

It is conceivable, further, that in a still rarer group of cases the infection might become an even more acute and generalized one.

Such a condition we have in the disease variously designated phlegmonous gastritis, mycotic gastritis, purulent inflammation of walls of stomach, submucous abscess, gastritis purulenta, suppurative gastritis, gastritis bacillaris, gastritis mycotica.

On this subject I can refer you to an admirably full account given in the *Edinburgh Hospital Reports* for 1896, by Dr. R. F. C. Leith.

It may be said that such a rare condition as phlegmonous gastritis can have little in common with such a comparatively common condition as that we are discussing,—viz., subacute and chronic gastric catarrh.

One might equally well maintain that such severe and generalized conditions as pyæmia and ulcerative endocarditis, or such intense local infections as acute osteomyelitis, cannot have the same underlying cause,—viz., pyogenic organisms as the smallest furuncle, the slightest local erysipelatous attack, or, lastly, as the suppurative process going on around a diseased tooth.

But, nevertheless, such is the case. The difference is not one of kind, but one of resistance and dose. If, as the above case would appear to show, some forms of subacute gastric conditions met with in association with suppurations around teeth may be due to infection of the mucosa by the pyogenic organisms swallowed, it may well be that from time to time the infection of the stomach wall may take on a specially virulent character. While the ordinary effect of infection underlying dental caries is to set up at most a slight local periostitis or a localized gum-boil, in certain cases the same condition may give rise to the most intense local suppurations or even give rise to general pyæmia.

In six out of the fifty-two cases of phlegmonous gastritis collected and tabulated by Dr. Leith, micro-organisms were actually found. "If the methods for detection of bacteria had been as well known as they now are, I have no doubt they would have been found in them all" (Leith).

In the case described by himself and in three of the above six the streptococcus was the chief organism present.

He draws attention to the parallelism betwixt phlegmonous gastritis and erysipelas, and notes that while we often cannot tell how or why an erysipelas arises, we never speak of it as being an obscure disease, whereas every author who has yet written upon phlegmonous gastritis has done so.

Leith thinks it may be looked upon as a severe form of erysipelas of the stomach.

Curiously enough, however,—and this is the interesting point in relation to our subject to-night,—in discussing the probable source of the infection in such cases, the various possibilities are considered to be two,—viz., the disease may arise from the side of the *stomach* or from the *blood.*

As to the conditions which favor the determination of the streptococcus to the stomach, the relative importance of alcoholic excess, dietetic errors, and overloading of stomach is considered. There is no suggestion that the source of infection might possibly be the mouth.

And yet one of the cases (No. 45), in which the symptoms came on six days after a tooth-extraction and death ensued on the tenth day, might well have had such an origin.

Condition found: In stomach submucous, muscular, and subserous coats infiltrated with pus; mucosa slightly hyperæmic; in mouth "the gums swollen, and showed purulent ulcers; alveoli of jaw slightly splintered. Submaxillary glands swollen."

The relation of events in this case I should conceive to be: a diseased tooth with purulent ulcers around as focus of infection; extraction; further inflammation and necrosis; constant flow of pus organisms into stomach for six days (probably also for a long period *before* the tooth was extracted); acute infection of its walls; suppurative inflammation and death.—*Transactions of the Odontological Society of Great Britain.*

